# Supplementary material for: Tunable translational control using site-specific unnatural amino acid incorporation in Escherichia coli
Source: PeerJ. 2015 Apr 28;3:e904. doi: 10.7717/peerj.904 (PMC4419535; doi:10.7717/peerj.904)
Supplement: Figure S1 — The amber-inserted EGFP expression sequence was cloned into pDONR221 using a standard Gateway reaction by BP recombination. [file peerj-03-904-s001.pdf]

1 ACAAGTTTGT ACAAAAAAGC AGGCTATCAA AAAAATATTC TCAACATAAA

attL1(3′) P_{lpp}

51 AAACTTTGTG TAATACTTGT AACGCTGCCA TCAGACGCAT TTCGAAGGAG

Shine-Dargarno sequence

101 ATAGAACCAT GTAGGAAGGT AAGCCTATCC CTAACCCTCT CCTCGGTCTC

Translation start Amber V5 epitope

151 GATTCTACGG TGAGCAAGGG CGAGGAGCTG TTCACCGGGG TGGTGCCCAT

EGFP gene

201 CCTGGTCGAG CTGGACGGCG ACGTAAACGG CCACAAGTTC AGCGTGTCCG

251 GCGAGGGCGA GGGCGATGCC ACCTACGGCA AGCTGACCCT GAAGTTCATC

301 TGCACCACCG GCAAGCTGCC CGTGCCCTGG CCCACCCTCG TGACCACCCT

351 GACCTACGGC GTGCAGTGCT TCAGCCGCTA CCCCGACCAC ATGAAGCAGC

401 ACGACTTCTT CAAGTCCGCC ATGCCCGAAG GCTACGTCCA GGAGCGCACC

451 ATCTTCTTCA AGGACGACGG CAACTACAAG ACCCGCGCCG AGGTGAAGTT

501 CGAGGGCGAC ACCCTGGTGA ACCGCATCGA GCTGAAGGGC ATCGACTTCA

551 AGGAGGACGG CAACATCCTG GGGCACAAGC TGGAGTACAA CTACAACAGC

601 CACAACGTCT ATATCATGGC CGACAAGCAG AAGAACGGCA TCAAGGTGAA

651 CTTCAAGATC CGCCACAACA TCGAGGACGG CAGCGTGCAG CTCGCCGACC

701 ACTACCAGCA GAACACCCCC ATCGGCGACG GCCCCGTGCT GCTGCCCGAC

751 AACCACTACC TGAGCACCCA GTCCGCCCTG AGCAAAGACC CCAACGAGAA

801 GCGCGATCAC ATGGTCCTGC TGGAGTTCGT GACCGCCGCC GGGATCACTC

851 TCGGCATGGA CGAGCTGTAC AAGTAAGACC CAGCTTTCTT GTACAAAGTG

attL2(5′)
