# Supplementary figures and images for: Tunable translational control using site-specific unnatural amino acid incorporation in Escherichia coli
Source: PeerJ. 2015 Apr 28;3:e904. doi: 10.7717/peerj.904 (PMC4419535; doi:10.7717/peerj.904)

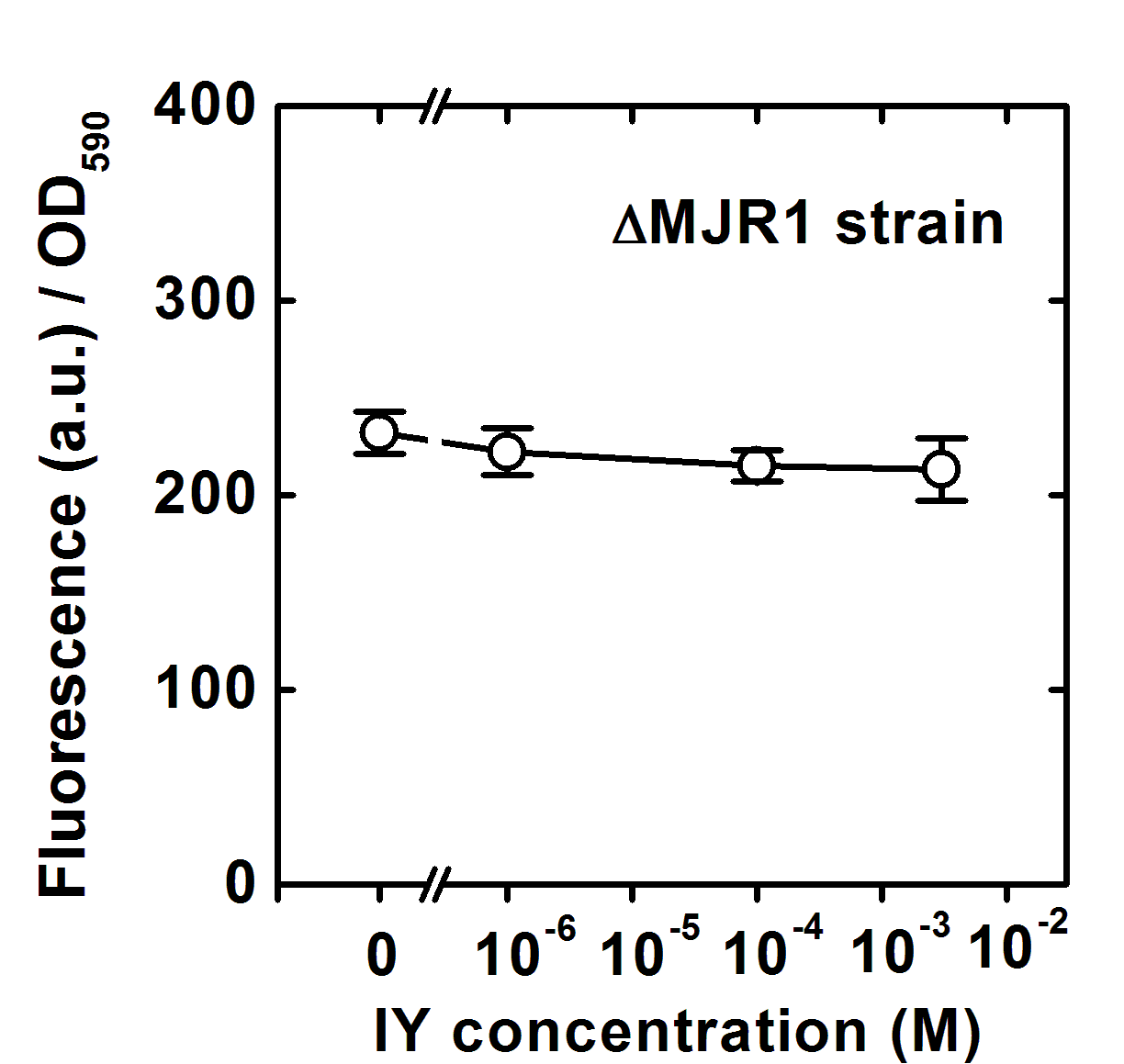

Supplement: Figure S2 — A bacterial strain carrying both the ΔMJR1 and the amber-inserted EGFP expression plasmid was evaluated. EGFP fluorescence was measured at various IY concentrations. Data are shown as mean ± SEM. n = 3 independent experiments. Statistical analysis was performed using Welch’s t-test (α = 0.05). No significant differences were detected in EGFP fluorescence intensity. [file peerj-03-904-s002.png]

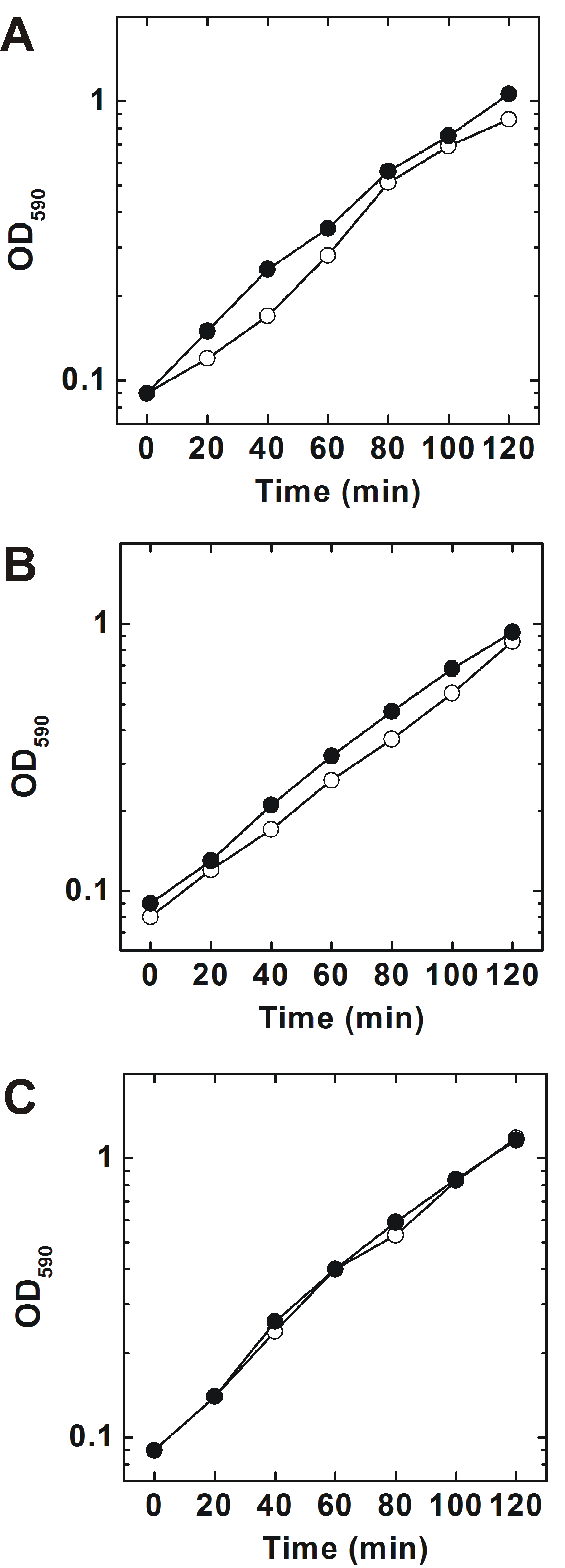

Supplement: Figure S3 — (A) pTYR MjIYRS2-1(D286) MJR1 × 3 and the amber-inserted EGFP expression plasmid. (B) pTYR MjIYRS2-1(D286) MJR1 × 3 alone. (C) The parental strain BL21-AI without any plasmids. Open circle, 0 M; filled circle, 3 × 10^−3^ M. [file peerj-03-904-s003.png]
